# SBL-JP-0004: A promising dual inhibitor of JAK2 and PI3KCD against gastric cancer

**DOI:** 10.32604/or.2024.055677

**Published:** 2024-12-20

**Authors:** HASSAN M. OTIFI

**Affiliations:** Department of Pathology, College of Medicine, King Khalid University, Abha, 62521, Saudi Arabia

**Keywords:** Gastric cancer (GC), Dual inhibition, JAK2, PI3KCD, SBL-JP-0004, Molecular docking, Molecular dynamics simulation

## Abstract

**Background:**

Gastric cancer (GC) remains a global health burden and is often characterized by heterogeneous molecular profiles and resistance to conventional therapies. The phosphoinositide 3-kinase and PI3K and Janus kinase (JAK) signal transducer and activator of transcription (JAK-STAT) pathways play pivotal roles in GC progression, making them attractive targets for therapeutic interventions.

**Methods:**

This study applied a computational and molecular dynamics simulation approach to identify and characterize SBL-JP-0004 as a potential dual inhibitor of JAK2 and PI3KCD kinases. KATOIII and SNU-5 GC cells were used for *in vitro* evaluation.

**Results:**

SBL-JP-0004 exhibited a robust binding affinity for JAK2 and PI3KCD kinases, as evidenced by molecular docking scores and molecular dynamics simulations. Binding interactions and Gibbs binding free energy estimates confirmed stable and favorable interactions with target proteins. SBL-JP-0004 displayed an half-maximal inhibitory concentration (IC_50_) value of 118.9 nM against JAK2 kinase and 200.9 nM against PI3KCD enzymes. SBL-JP-0004 exhibited potent inhibition of cell proliferation in KATOIII and SNU-5 cells, with half-maximal growth inhibitory concentration (GI_50_) values of 250.8 and 516.3 nM, respectively. A significant elevation in the early phase apoptosis (28.53% in KATOIII cells and 26.85% in SNU-5 cells) and late phase apoptosis (17.37% in KATOIII cells and 10.05% in SNU-5 cells) were observed with SBL-JP-0004 treatment compared to 2.1% and 2.83% in their respective controls.

**Conclusion:**

The results highlight SBL-JP-0004 as a promising dual inhibitor targeting JAK2 and PI3KCD kinases for treating GC and warrant further preclinical and clinical investigations to validate its utility in clinical settings.

## Introduction

Chronic inflammation of the gastric epithelium can lead to stomach or gastric cancer (GC) in some individuals [1–3]. More than 90% of GCs are adenocarcinomas originating from the chronically inflamed gastric mucosal epithelium. Less common forms of GC include lymphoma of the stomach, gastrointestinal stromal tumors, and gastrointestinal carcinoid tumors [4,5]. Other major contributors to GC include genetic mutations, chromosomal abnormalities, and gene aberrations at the molecular level. Identifying biomarkers and molecular pathways associated with GC is crucial for early detection and intervention. Modern molecular classifications highlight the significant roles of several signaling pathways, such as RAS/RAF/MAPK, HER2, PI3K/JAK2/mTOR, NFκB, JAK/STAT, c-MET, P53, and TGF-β [6,7].

The phosphoinositide-3 kinase (PI3K) pathway is particularly important in GC tumorigenesis, as it contributes to drug resistance, promotes angiogenesis, inhibits apoptosis, and accelerates metastasis [8]. PI3K has three classes: I, II, and III. Class I is further divided into IA and IB, with the catalytic subunit p110 of class IA comprising four isoforms: p110α, p110β, p110γ, and p110CD, encoded by the genes PI3KCA, PI3KCB, PI3KCG, and PI3KCD, respectively [9]. While PI3KCA is largely implicated in solid tumors such as GC, PI3KCD, traditionally associated with hematological cancers, has recently garnered interest for its role in solid tumors, including GC, due to its immunomodulatory effects and impact on cell-mediated tolerance to cancer [10]. The other types of PI3Ks include PI3K alpha and gamma, PI3Kα is a class I PI3K primarily involved in cell growth, and PI3Kγ plays a vital role in chemokine-dependent migration of neutrophils and macrophages [11].

Given that GC often arises from inflammation of the gastric mucosa, the role of cell communication in response to external stimuli and ensuing inflammation is critical. This communication is mediated by signaling molecules, including cytokines such as interleukins, chemokines, interferons, and tumor necrosis factors, which activate key pathways like the Janus Kinase (JAK), Signal Transducer and Activator of Transcription (STAT)—JAK/STAT signaling pathway [4]. This pathway regulates immune response, cell proliferation, survival, and apoptosis. Genetic abnormalities or external factors can disrupt normal regulation, leading to the initiation and progression of tumors [12].

Treatment options for GC patients include surgery, chemotherapy, and radiotherapy. However, chemoresistance often develops due to survival signals triggered by receptors activated through the PI3K pathway, which inhibit apoptosis [9,13]. Targeting both JAK2 and PI3KCD simultaneously can potentially overcome the limitations of single-agent therapies by blocking multiple survival and proliferation signals, thereby reducing tumor growth and metastasis [14]. As there are no known drugs approved for drugs targeting both JAK2 and PI3KCD (dual inhibitors), targeting these two pathways with separate molecules may lead to more cytotoxicity, as kinase inhibitors pose several side effects. Dual inhibition may also mitigate the development of drug resistance, a common challenge in cancer treatment. This approach shows significant potential for treating GC, where the tumor’s heterogeneous nature and the interaction of multiple signaling pathways require a multifaceted therapeutic strategy. Therefore, better strategies are needed to overcome these limitations. Given the heterogeneous nature of gastric tumors and the limited efficacy of chemotherapy, this study focuses on identifying dual inhibitors targeting both JAK and PI3KCD pathways.

## Materials and Methods

### Materials

Reagents and chemicals were procured from Sigma Aldrich (St. Louis, MO, USA. The reagents, and buffers used in this study for the biochemical analysis were purchased from Sigma Aldrich (St. Louis, MO, USA) unless specified). The Vero (CCL-81), KATO III (HTB-103), and SNU-5 (CRL-5973) cell lines were obtained from the American Type Culture Collection (ATCC, Rockville, MD, USA). The PI 3-Kinase (human) HTRF enzyme assay kit (#33-047) was sourced from UPSTATE, Merck Millipore (Burlington, MA, USA). The luminescence-based JAK2 assay kit (#79520) was acquired from BPS Bioscience (San Diego, CA, USA). The Annexin V assay kit (#CBA059) was provided by Merck Millipore (Burlington, MA, USA). Lead compound SBL-JP-0004 (catalog number # 5162323) was purchased from ChemBridge Corporation (San Diego, CA, USA).

### Methods

#### Structure retrieval and high-throughput virtual screening

The theoretical model of JAK2 was retrieved from the AlphaFold database (https://alphafold.ebi.ac.uk/, accessed on 12 November 2024), and the experimental structure of PI3KCD was obtained from the PDB databank (https://www.rcsb.org/structure/5UBT, accessed on 12 November 2024). Before docking, these structures were processed using Discovery Studio Visualizer (2017 R2), involving removing crystal waters and adding polar hydrogens. Any missing side chains and atoms were addressed using the SwissPDB viewer (4.1.0). Docking preparations included setting up a docking grid box centered on the reference ligand of each target protein, with dimensions of 20 units on all sides. High-throughput virtual screening of the ChemBridge library (https://www.hit2lead.com/, accessed on 12 November 2024), focusing on molecules with a molecular weight (MW) between 350 to 750 Da, was performed using the D-HTVS module from SiBioLEAD (https://sibiolead.com/, accessed on 12 November 2024). Protein-ligand interactions were subsequently analyzed using the PLIP analysis plugin (V 2.2.0), and the results were visualized using Discovery Studio Visualizer.

#### Molecular dynamic simulations

The Molecular Dynamics (MD) simulation of the protein-ligand complex was performed using the GROMACS simulation package (V2021) accessed via the MD simulation server hosted by SiBioLEAD, accessed via https://sibiolead.com/ (accessed on 12 November 2024) [15]. The ligand topology was generated using AMBERTOOLS and ACPYPE (V 2022.7.21), and system parameterization was achieved with the OPLS/AA force field. The protein-ligand complex was initially placed in a triclinic box filled with Simple Point Charge (SPC) water molecules for hydration. The simulation system was neutralized with NaCl counterions to mimic physiological conditions and supplemented with 0.15M NaCl salt concentration. The system underwent energy minimization using the steepest descent method over 5000 steps. Subsequently, equilibration was conducted for 300 ps under constant temperature (NVT) and pressure (NPT) conditions. The MD simulation was run for 100 nanoseconds (ns) using a leap-frog integrator, with trajectory frames recorded every 10 ps. Trajectory analysis utilized built-in GROMACS scripts, and results were visualized using xmgrace (V 5.1.25).

Protein preparation → Energy minimizations → equilibration final MD → trajectory analysis.

#### MMPBSA-based binding energy calculations

The Gibbs binding free energy (Δ*G*_binding_) of the protein-ligand complexes was calculated using the GMX_MMPBSA method [16]. This involved analyzing trajectory frames saved at 2 ns intervals throughout the entire 100 ns simulation period to compute the ΔG binding values.

#### JAK2 enzyme inhibition assay

The assay was performed using a luminescence-based JAK2 assay kit (Catalog #79520) from BPS Bioscience, CA, USA, following the manufacturer’s instructions. In brief, 25 μL of master mix in distilled water, which included kinase assay buffer with 1 M Dithiothreitol (DTT), 500 µM ATP, and Protein Tyrosine Kinase Substrate (poly-Glu, Tyr 4:1), was prepared. To this mix, 5 μL of the test compound at varying concentrations (0.1 to 3000 nM) was added to a 96-well plate. The reaction was initiated by adding 2.5 ng/μL JAK2 enzyme and incubating at 30°C for 45 min. Subsequently, 50 μL of Kinase-Glo MAX (Promega Corporation, Madison, WI, USA #V6071) was added to each well and incubated at room temperature for 15 min. Luminescence was recorded using a FLUOstar Omega plate reader (BMG Labtech, Cary, NC, USA). IC_50_ values were calculated using GraphPad Prism software (version 6.0).

#### PI3KCD enzyme assay

The assay was carried out using PI 3-Kinase (human) HTRF assay using UPSTATE kit (# catalog no. 33-016) from Millipore using PI3-KCD isoform. Briefly, 14.5 μL of PI3KCD/PIP2 mix in 1X reaction buffer supplied from the kit, was added to plus enzyme control wells and inhibitor test wells. The minus enzyme control wells were added with 14.5 μL 1X reaction buffer. All control wells were added with 0.5 μL of DMSO, while inhibitor test wells were added with different concentrations (0.1 to 3000 nM) of inhibitors in 0.5 μL of DMSO. All wells were treated with 5 μL of ATP and incubated for 30 min at room temperature. The reaction was arrested with 5 μL of stop solution followed by a 5 μL detection mix. The plate was incubated at room temperature for overnight in the dark. The HTRF ratio was measured at excitation—337 nm, emission 615 and 665 nm, counting delay 50 µsec, counting window 400 µsec. IC_50_ values were calculated using GraphPad Prism software (version 6.0).

#### Cell proliferation assay

Cancer and Vero cells were cultured in a complete growth medium under standard conditions. According to conventional procedure, Vero (CCL-81), KATO III (HTB-103), and SNU-5 (CRL-5973) cell lines were cultured in (DMEM, Sigma Aldrich St. Louis, MO, USA # D5796) with fetal bovine serum (10% FBS), 100 U/mL of penicillin, and 100 U/mL of streptomycin. Assays were conducted when cells reached 80% confluency. The cells were microscopically examined for mycoplasma or any contamination and tested for reproducibility of results. Cell proliferation was measured using the 3-(4, 5-dimethylthiazolyl-2)-2, 5-diphenyltetrazolium bromide (MTT) assay as previously described [17]. KATOIII or SNU-5 cells (5 × 10^3^ cells/well) were plated in 96-well tissue culture plates with a regular growth medium. The cells were treated with various concentrations (1 to 10000 nM) of SBL-JP-0004 for 48 h. After removing the medium, the cells were incubated with 100 μL MTT solution (1 mg/mL) for 4 h. The resulting formazan products were dissolved in 200 μL of DMSO, and absorbance was measured at 560 nm. Percent inhibition was calculated using GraphPad Prism 6.0 to determine the GI_50_ values.

#### Apoptosis analysis by annexin V assay

Apoptosis was evaluated using an Annexin V detection kit according to the manufacturer’s instructions. KATOIII or SNU-5 cells (0.5 × 10^6^) were seeded in 6-well plates and treated with the desired concentrations of SBL-JP-0004, followed by incubation in 5% CO_2_ at 37°C for 48 h. After incubation, the cells were harvested, washed with kit buffer, and incubated with 0.25 µg/mL Annexin V reagent for 15 min in the dark. After two washes with 1 × PBS, the cells were resuspended in a kit buffer containing 0.5 µg/mL propidium iodide. Data from ten thousand events were acquired using a Guava easyCyte flow cytometer (Guava easyCyte™ HT System, Merk Millipore, MA, USA), and analysis was performed with InCyte software (v.2.7, Merk Millipore, MA, USA) to differentiate between healthy and apoptotic cells (early and late apoptosis). Results were presented using GraphPad Prism software (version 6.0; La Jolla, CA, USA).

### Statistical analysis

Statistical analyses were made with GraphPad Prism 6.0. software (version 6.0; La Jolla, CA, USA). Results are expressed as mean ± SD. Data were analyzed using way ANOVA. Statistical significance was set at *p* < 0.05. GI_50_ and IC_50_ were calculated with linear regression analysis.

## Results

### High-throughput virtual screening identified small molecules that target JAK2 and PI3KCD

**Figure 1:**
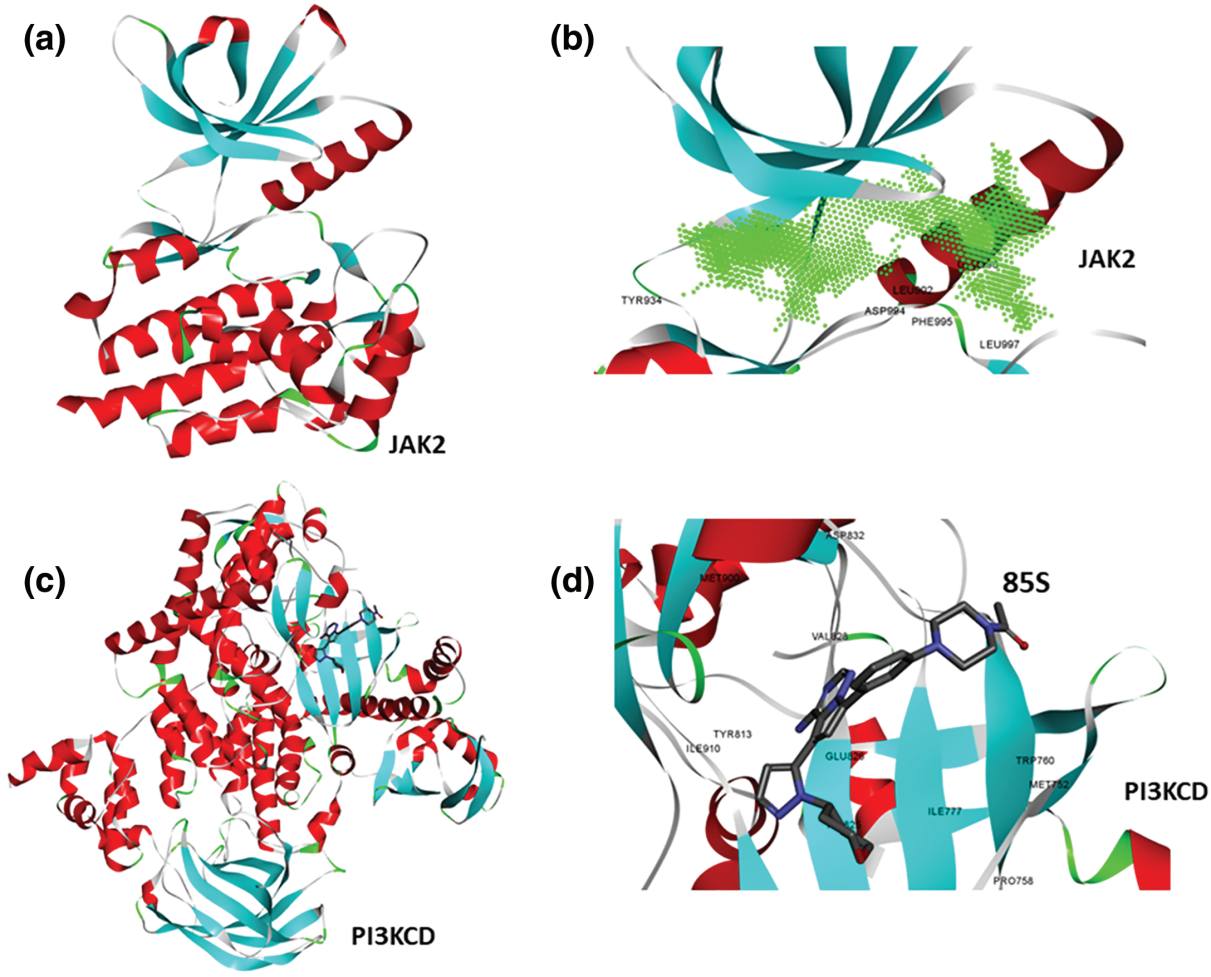
Structure of JAK2 and PI3KCD. It reveals druggable pocket. (a) Theoretical structure of JAK2 kinase, colored based on secondary structure. (b) Presence of ATP binding pocket (active site) kinase domain of JAK2, green contour representation, showing amino acid residues surrounding the cavity. (c) Crystal structure of PI3KCD kinase, colored based on secondary structure, bound to a reference ligand (85S). (d) protein-ligand interaction analysis showing key amino acid residues of PI3KCD involved in the interactions with 85S.

To identify small molecules targeting both JAK and PI3K pathways, the full-length structure of JAK2 kinase was predicted using AlphaFold, while the experimental structure of PI3KCD was retrieved from the PDB databank (PDB ID: 5UBT). Visualization of the JAK2 structure confirmed the completeness of the kinase domain (Fig. 1a). Using Discovery Studio Visualizer, a protein cavity/druggable site was predicted, revealing a large cavity at the kinase domain corresponding to the ATP binding pocket (Fig. 1b). Analysis of the active conformation of PI3KCD, bound to the reference ligand 85S, identified the key drug-binding site for inhibiting kinase activity (Fig. 1c). Further protein-ligand analysis highlighted critical amino acid residues involved in interactions with the reference ligand 85S (Fig. 1d). Based on these structural evaluations, the kinase domain of JAK2 and the active site of PI3KCD were selected for high-throughput virtual screening.

**Figure 2:**
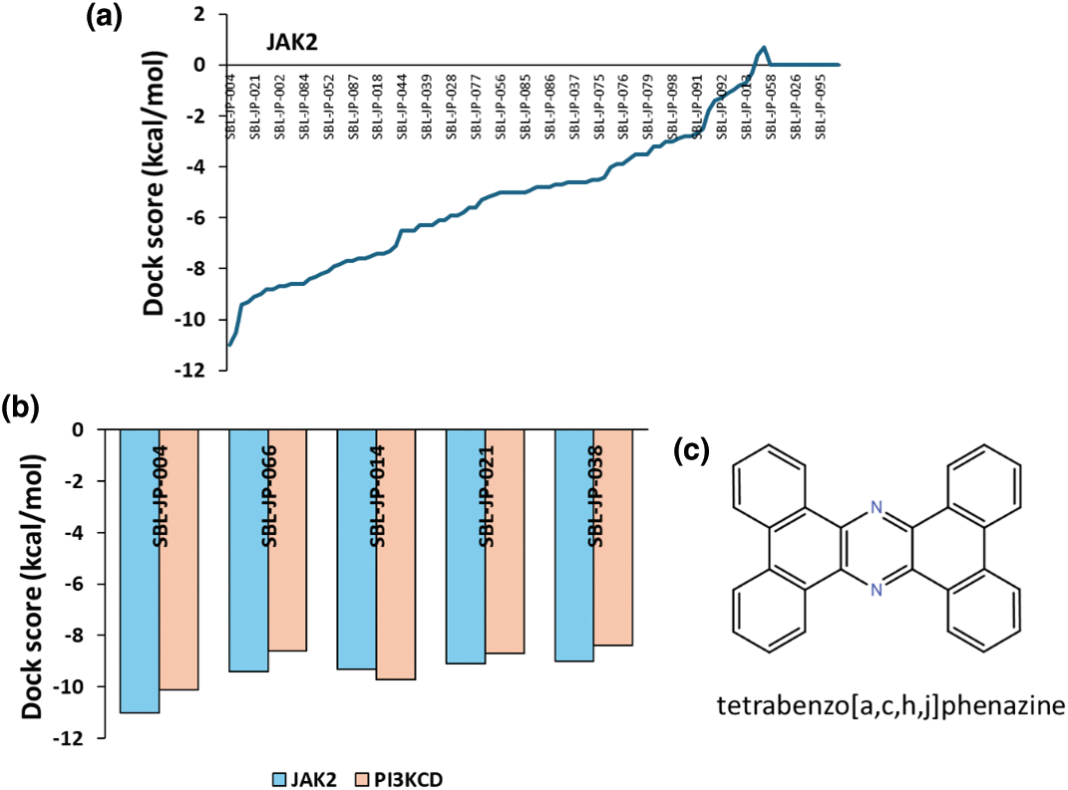
High-throughput Virtual Screening of ChemBridge library. (a) Predicted docking scores based on high-throughput Virtual Screening of ChemBridge library against JAK2 kinase domain, showing top-ranked ligands. (b) predicted docking scores for molecules binding to both JAK2 and PI3KCD kinase. (c) 2D structure of SBL-JP-004.

Identifying small molecules with high affinity for both JAK2 and PI3KCD kinases was carried out through a two-stage virtual screening process using the ChemBridge small molecule library. The entire ChemBridge library was screened against the JAK2 kinase domain in the first stage. A docking box was constructed around the kinase domain of JAK2, and a diversity-based, high-throughput virtual screening approach was implemented via the Autodock-Vina package on the SiBioLEAD server. The top compounds were ranked based on their docking scores (Fig. 2a). The top 100 compounds with high affinity for JAK2 kinase were subjected to a second-stage screening against the PI3KCD kinase, focusing on the reference ligand 85S binding region. Compounds were ranked again based on docking scores, revealing that compound SBL-JP-0004 (tetrabenzo[a,c,h,j]phenazine) from the ChemBridge library exhibited high affinity for both JAK2 and PI3KCD kinases (Fig. 2b).

### Protein-ligand interaction analysis shows SBL-JP-0004 as a potent dual inhibitor

**Figure 3:**
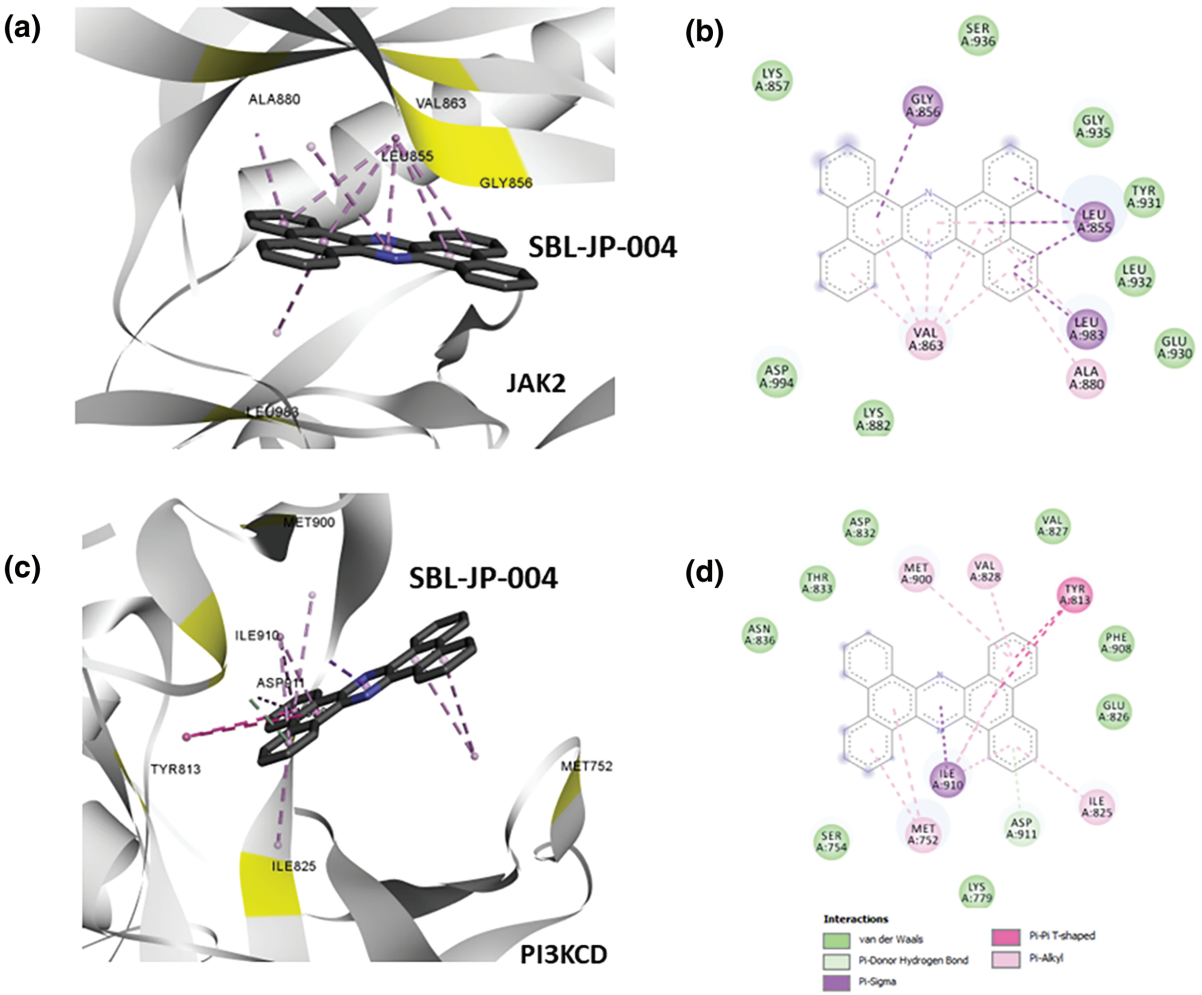
Protein-ligand interaction analyses. (a) Predicted binding pose for SBL-JP-0004 with JAK2. (b) Protein-ligand interaction analysis profiling shows amino acid residues of JAK2 in contact with the lead molecule SBL-JP-0004. (c) Predicted binding pose for SBL-JP-0004 with PI3KCD kinase. (d) Protein-ligand interaction analysis profiling shows amino acid residues of PI3KCD kinase in contact with the lead molecule SBL-JP-0004.

The protein-ligand interaction profiler from Discovery Studio Visualizer was used to analyze the number and strength of interactions between the ligands and the target kinases. The results showed that SBL-JP-0004 exhibited interaction energies of −11.0 and −10.1 for JAK2 and PI3KCD kinases, respectively (Fig. 3). For JAK2, SBL-JP-0004 formed several pi-pi stacking interactions with critical amino acid residues in the kinase domain, including Gly856, Leu855, Leu983, and Val863 (Fig. 3a,b). The analysis also indicated that SBL-JP-0004 fits well within the ATP binding region of JAK2 kinase. Similarly, SBL-JP-0004 strongly binds to PI3KCD, forming more than eight pi-pi stacking interactions. The compound fits well within the target site of PI3KCD, interacting with residues such as Met300, Val828, Tyr813, and Met752 (Fig. 3c,d). Based on these interactions, SBL-JP-0004 was selected as a lead candidate for further analysis.

### Molecular dynamic simulation showed SBL-JP-0004 binds stably to JAK2 and PI3KCD

To further elucidate the stability of ligand binding and understand the dynamic behavior of the protein-ligand complexes, atomistic MD simulations of the target kinases bound to the predicted dual inhibitor, SBL-JP-0004, were conducted. Using the GROMACS simulation package via the SiBioLEAD web server (www.sibiolead.com, accessed on 12 November 2024), 100 ns MD simulations were performed for each complex.

**Figure 4:**
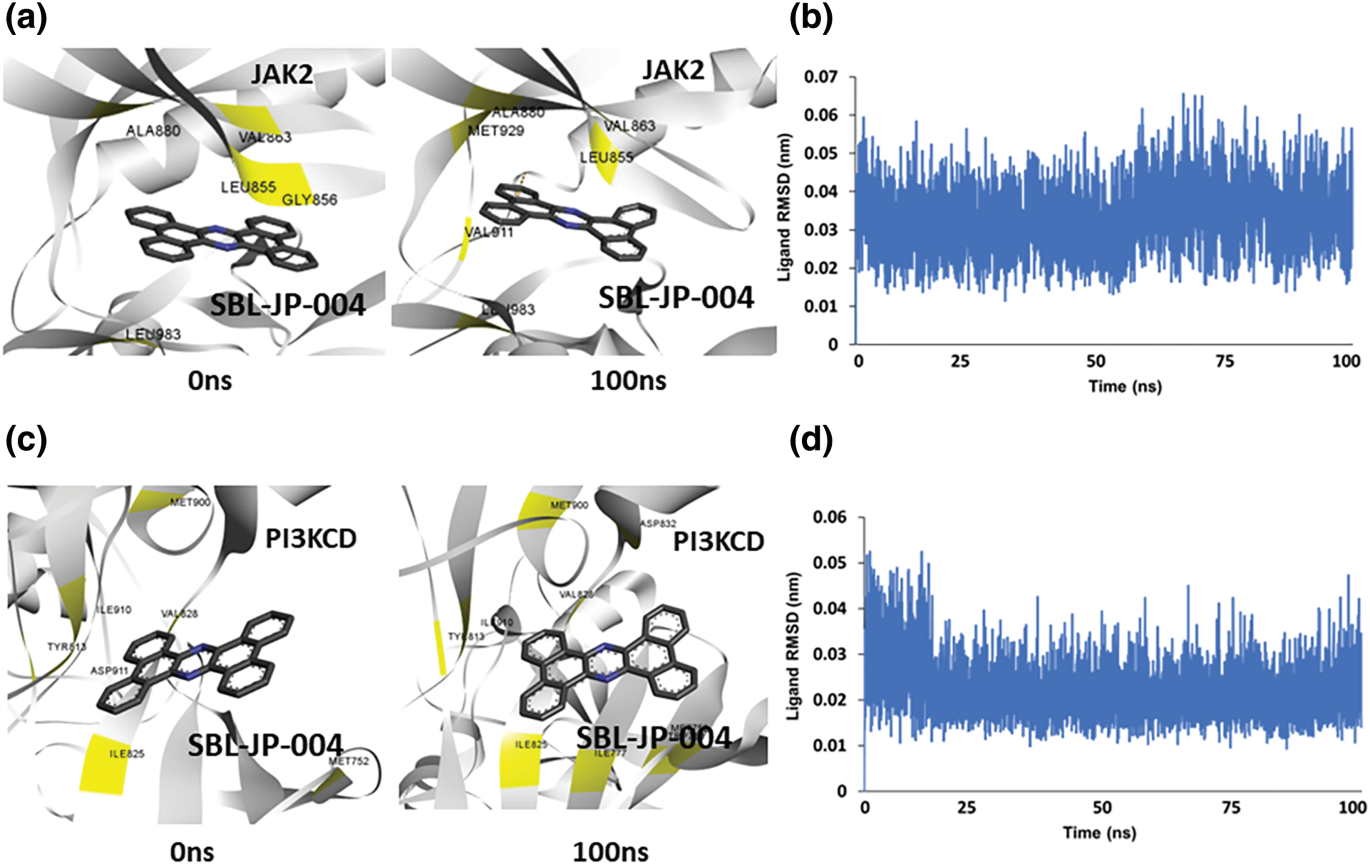
Molecular dynamic simulation predicts protein-ligand stability. (a) Snapshot of simulation trajectories captured before and after 100 ns simulation, depicting SBL-JP-0004 binding to JAK2. (b) Ligand Root Mean Square Deviation (RMSD) of SBL-JP-0004 in complex with JAK2, depicting binding stability. (c) Snapshot of simulation trajectories captured before and after 100 ns simulation, depicting SBL-JP-0004 binding to PI3KCD kinase. (d) Ligand Root Mean Square Deviation (RMSD) of SBL-JP-0004 in complex with PI3KCD kinase, depicting the binding stability.

Initially, the SBL-JP-0004::JAK2 complex was immersed in a triclinic box containing SPC water and counterions. After energy minimization and equilibration, the system was simulated for 100 ns using a leapfrog integrator. Simulation trajectories were analyzed to assess binding stability. Comparative analysis of snapshots taken at 0 and 100 ns of the simulation revealed consistent stability of SBL-JP-0004 binding to JAK2 kinase throughout the simulation (Fig. 4a). Evaluation of the ligand’s spatial arrangement at these time points provided insights into the dynamic nature of the complex. The interacting amino acid residues remained unchanged, indicating stable binding. Furthermore, the ligand Root Mean Square Deviation (RMSD) was calculated to gauge binding stability over time. The RMSD remained stable, averaging around 0.03 nm, indicating overall stability and equilibrium of SBL-JP-0004 in the complex with JAK2 (Fig. 4b).

Similarly, the predicted SBL-JP-0004::PI3KCD complex underwent a 100 ns MD simulation using the same protocol. Analysis of trajectory snapshots before and after the simulation indicated that SBL-JP-0004 bound stably and comfortably at the allosteric site in PI3KCD kinase (Fig. 4c). Notably, after the 100 ns simulation, the number of interacting amino acid residues with SBL-JP-0004 increased, suggesting a significant binding affinity. Ligand RMSD showed a stable plateau throughout the simulation, indicating stable binding (Fig. 4d). This observation further supports the notion of stable and robust binding of SBL-JP-0004 to both JAK2 and PI3KCD kinases, underscoring its potential as a dual inhibitor for treating GC.

**Figure 5:**
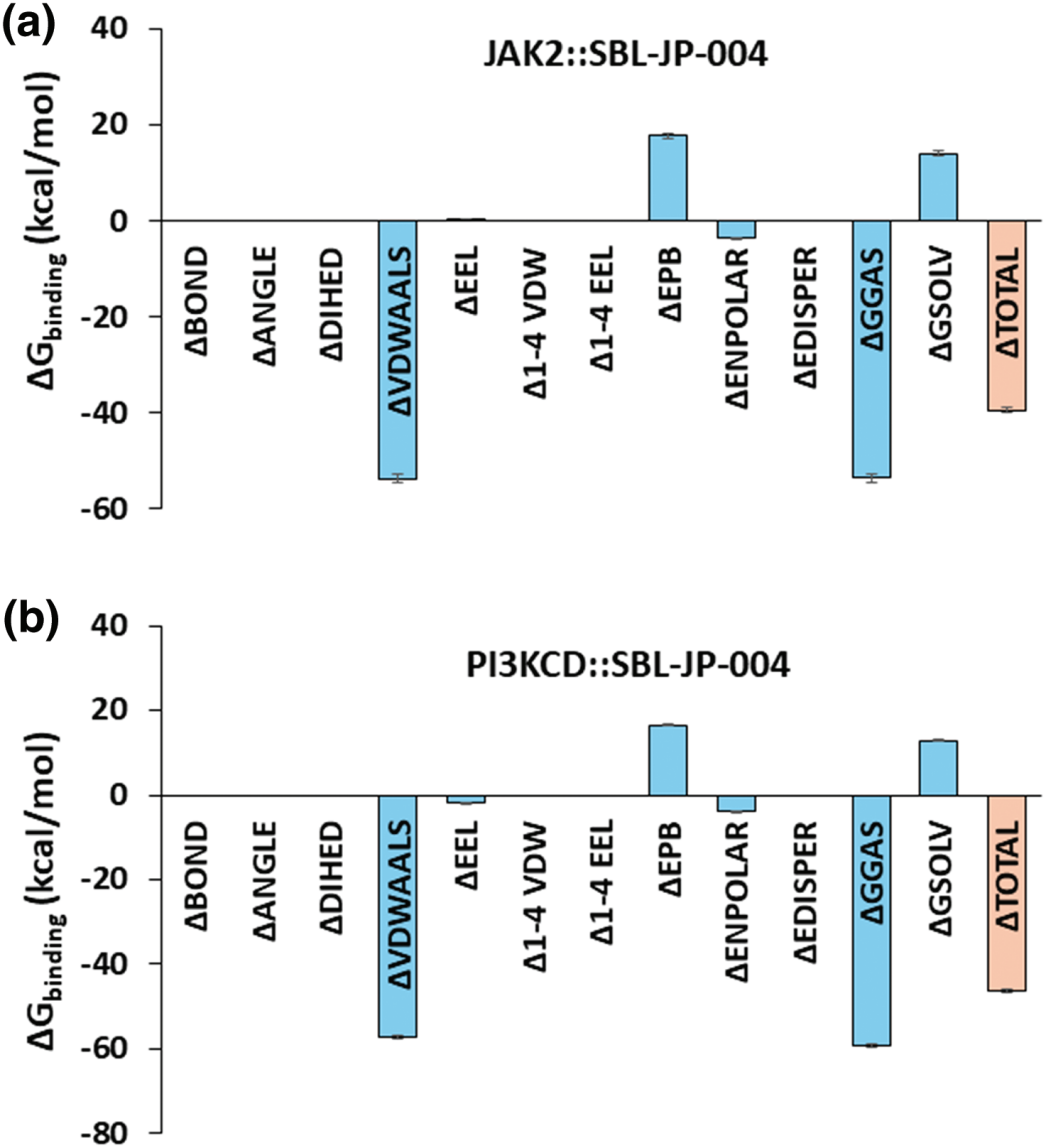
Binding free-energy estimation. MM-PBSA-based binding free energy estimate shows the average Gibbs binding energy estimate between SBL-JP-0004 and (a) JAK2 and (b) PI3KCD kinase, calculated from 100 ns simulation trajectories.

To further assess the thermodynamic stability of the protein-ligand interactions in the SBL-JP-0004::JAK2 and SBL-JP-0004::PI3KCD kinase complexes throughout the 100 ns simulation trajectories, Molecular Mechanics Poisson-Boltzmann Surface Area (MM-PBSA) based binding free energy estimates were calculated. Fifty frames spanning the 100 ns simulation trajectories were selected for this analysis. Using the gmx_MMPBSA method, Δ*G*_binding_ for each of the 50 frames was computed. The results revealed that the average Δ*G*_binding_ between SBL-JP-0004 and JAK2 was −39.39 kcal/mol (Fig. 5a). For the SBL-JP-0004::PI3KCD complex, the Δ*G*_binding_ was −46.36 kcal/mol, indicating significant binding stability for the target molecule (Fig. 5b). These findings suggest robust and thermodynamically stable interactions between SBL-JP-0004 and both JAK2 and PI3KCD kinases.

### SBL-JP-0004 inhibited JAK2 and PI3KCD to control gastric cancer proliferation

**Figure 6:**
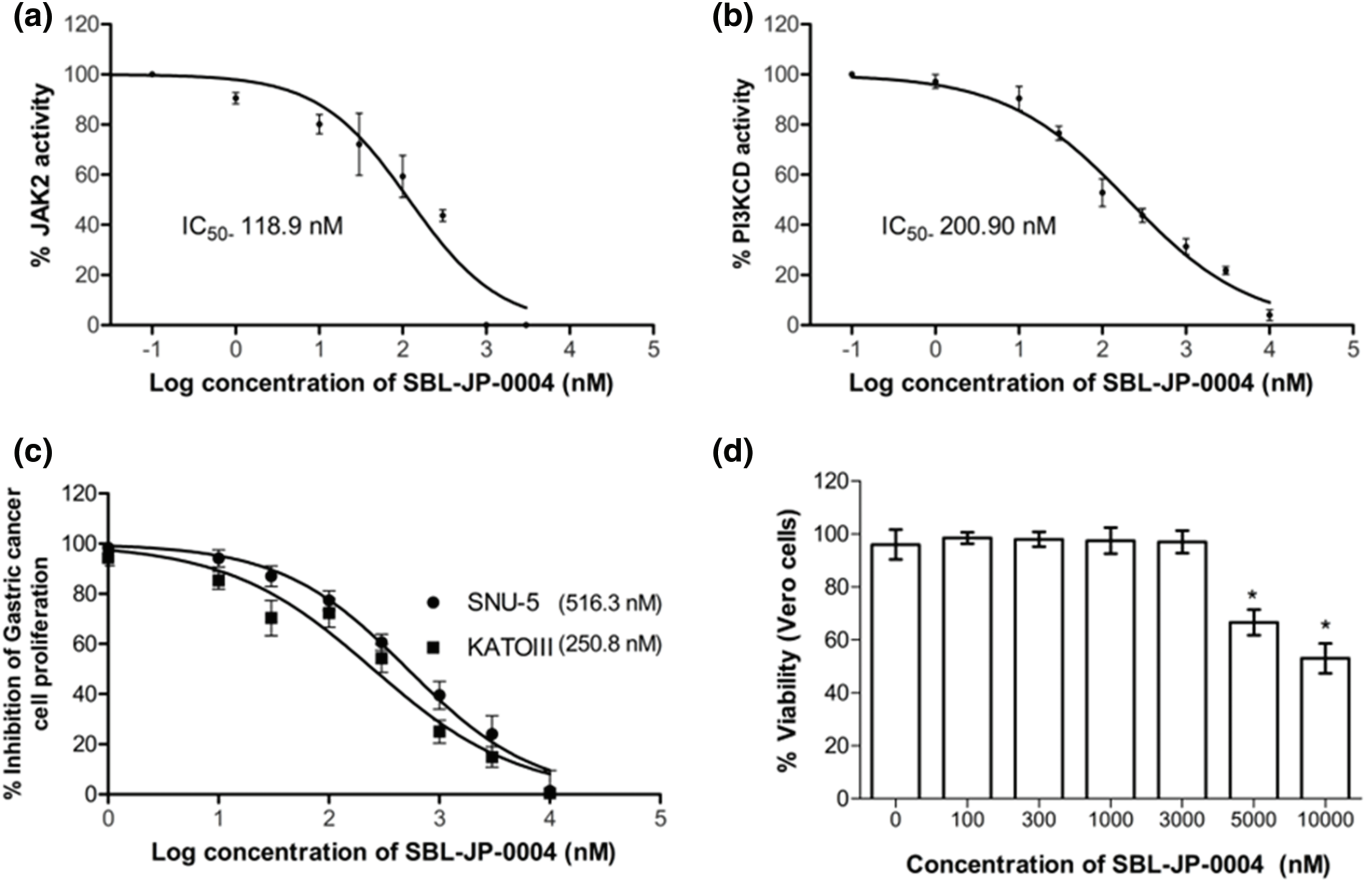
Biological efficacy of SBL-J2P-004. The enzyme IC_50_ values of SBL-JP-0004 against JAK2 (a) and PI3KCD (b) are shown. These values represent the mean ± standard deviation (SD) from three independent experiments, with IC_50_ values determined using GraphPad Prism version 6.0 software. Additionally, the GI_50_ values for cell proliferation in KATOIII and SNU-5 cells treated with SBL-JP-0004 (c), as well as the impact of the compound on the viability of Vero cells at various concentrations (d), are illustrated. Cell proliferation and viability were assessed using the MTT assay, with mean ± SD values analyzed using GraphPad Prism version 6.0 software, *p < 0.05.

To validate the computational predictions, *in vitro* enzyme inhibition studies were conducted for the JAK2 and PI3KCD enzyme targets. SBL-JP-0004 inhibited JAK2 and PI3KCD in a dose-dependent manner, with IC50 values of 118.9 and 200.90 nM, respectively (Fig. 6a,b). The efficacy of SBL-JP-0004 against GC cell proliferation was analyzed using the MTT assay. The compound inhibited the proliferation of both SNU-5 and KATOIII GC cells in a dose-dependent manner, with GI_50_ values of 516.3 and 250.8 nM, respectively (Fig. 6c). To assess the tolerability of these biologically active doses in normal, non-cancerous cells, various concentrations of SBL-JP-0004 were tested on Vero cell proliferation. The compound did not affect Vero cell proliferation up to 3000 nM (Fig. 6d).

### SBL-JP-0004 induced early and late phase apoptosis of the gastric cancer cells

**Figure 7:**
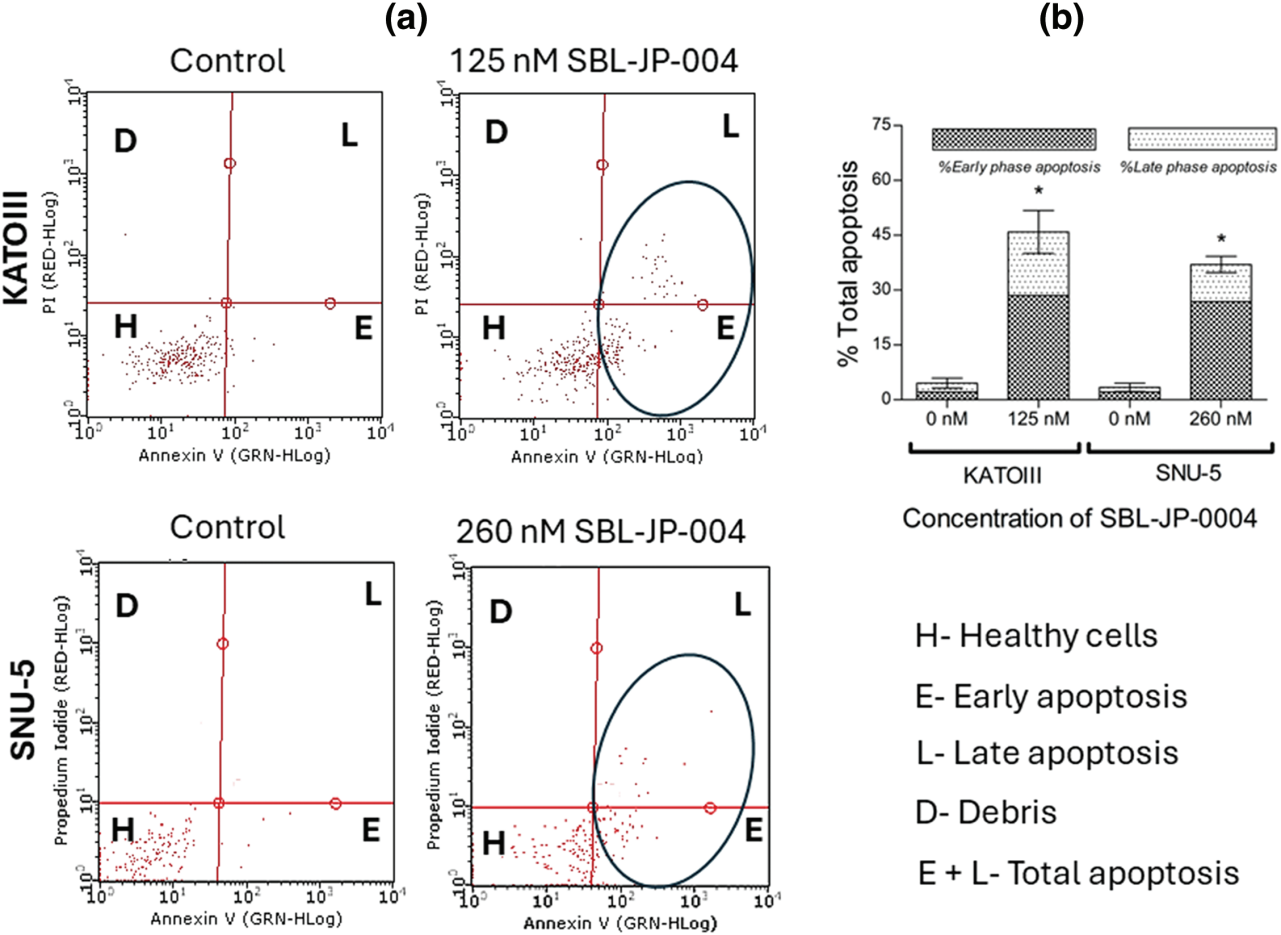
Early and late phase apoptosis induction by SBL-JP-004. The compound induced apoptosis: (a) Annexin V assay results showing the proportions of early and late apoptotic cells in KATOIII and SNU-5 cells following treatment with SBL-JP-0004 are presented. (b) The compound increased both early and late apoptotic populations in these cells after 48 h of treatment. Each experiment was performed in triplicate, and the results shown are representative. Data are expressed as mean ± SD. *p < 0.05.

To determine if the anti-proliferative efficacy of SBL-JP-0004 impacted other cellular functions in GC cells, an Annexin V flow cytometry assay was performed using concentrations near half of the GI_50_ values (125 nM for KATOIII and 260 nM for SNU-5 cells). Analysis of apoptosis revealed that treatment with SBL-JP-0004 increased the proportion of early and late apoptotic cells in both GC cell types (Fig. 7). Specifically, SBL-JP-0004 treatment elevated early apoptosis to 28.53% in KATOIII cells and 26.85% in SNU-5 cells, compared to 2.1% and 2.83% in their respective controls (Fig. 7). Regarding late-phase apoptosis, KATOIII cells had 17.37% positive population. In comparison, SNU-5 cells had a 10.05% positive population (Fig. 7).

## Discussion

The investigation of dual inhibition targeting JAK2 and PI3KCD in GC provides a promising therapeutic strategy aiming to address the complex and polygenic nature of this disease. GC is characterized by its heterogeneous pathology and resistance to conventional treatments and involves multiple signaling pathways that drive tumor progression, survival, and metastasis [18]. Two key pathways, JAK/STAT and PI3K, are especially important because they help control how cells grow, survive, and interact with the immune system. The proteins JAK2 and PI3KCD play a major role in these processes. Simultaneously targeting these two kinases, enhances therapeutic efficacy and overcomes the limitations of single-agent therapies, which often leads to resistance. Identifying SBL-JP-0004 as a dual inhibitor of JAK2 and PI3KCD holds significant promise for advancing the treatment of GC. This study demonstrated that SBL-JP-0004 exhibits strong binding affinity and stability with both JAK2 and PI3KCD, as predicted by both molecular docking studies and molecular dynamics (MD) simulations. The interaction energies and binding free energy estimates indicate stable and strong interactions, suggesting that SBL-JP-0004 can effectively inhibit the activity of both kinases [19]. This dual-targeted approach aims at blocking key pathways that drive GC, offering a way to get around the limits of current treatments. These treatments often do not work as well because GC is complicated and varied, leading to issues like drug resistance [20–22].

Molecular docking studies revealed that SBL-JP-0004 forms multiple strong interactions within the ATP binding pockets of JAK2 and PI3KCD. The protein-ligand interaction analysis showed that SBL-JP-0004 engages in significant pi-pi stacking interactions with key amino acid residues, ensuring stable and effective binding. These findings were further supported by MD simulations, which demonstrated consistent binding stability over the 100 ns simulation period. The low Root Mean Square Deviation (RMSD) values observed in both complexes indicate that SBL-JP-0004 maintains a stable conformation when bound to JAK2 and PI3KCD, reinforcing its potential as a dual inhibitor [23].

Furthermore, the thermodynamic stability assessed through MM-PBSA calculations provided additional insights into the efficacy of SBL-JP-0004. The binding free energy estimates for the SBL-JP-0004::JAK2 and SBL-JP-0004::PI3KCD complexes were significantly negative, indicating favorable binding interactions [24]. The consistent binding energy across multiple frames of the MD simulation suggests that SBL-JP-0004 can effectively inhibit these kinases under physiological conditions. This thermodynamic stability is crucial for the sustained inhibition of JAK2 and PI3KCD activities, which are often upregulated in GC and contribute to tumor growth, survival, and metastasis [25,26].

The computational findings of this study were validated by *in vitro* evaluations. SBL-JP-0004 effectively inhibited both JAK2 and PI3KCD enzyme activity, consistent with its anti-proliferative effects observed in GC cell assays. Notably, the compound demonstrated significant potency against GC cells while sparing normal Vero cells, as indicated by GI_50_ values that did not affect Vero cell viability. Previous studies have reported that inhibitors targeting both JAK2 and PI3KCD can induce apoptosis in GC cells [27]. These results highlight the potential of SBL-JP-0004 as a promising therapeutic agent for GC.

The dual inhibition of JAK2 and PI3KCD by SBL-JP-0004 offers a strategic advantage in treating GC. By concurrently targeting two critical pathways, this approach can disrupt multiple survival and proliferation signals within cancer cells [28], thereby enhancing the therapeutic efficacy and potentially reducing the likelihood of resistance development [29]. Additionally, this strategy may improve patient outcomes by addressing the diverse molecular drivers of GC [20]. Although the results from this study show promise in the strategy, these results are through computational modeling and *in vitro* studies which are the limitations, therefore, further *in vivo* evaluations, including efficacy, safety, and pharmacokinetics properties of the lead compound, SBL-JP-0004, are needed to move forward toward pre-clinical and clinical studies. Ultimately, the dual inhibition of JAK2 and PI3KCD represents a novel and potent therapeutic avenue for the treatment of GC, offering hope for better management of this challenging disease.

## Conclusion

In conclusion, the study highlights the promising potential of dual inhibition targeting JAK2 and PI3KCD as a novel therapeutic strategy for GC. SBL-JP-0004 has shown a strong and stable attachment to both kinases, backed by detailed computer simulations and energy calculations. The compound’s ability to effectively disrupt key pathways involved in tumor progression, survival, and metastasis highlights its therapeutic relevance in overcoming the challenges posed by gastric tumors’ heterogeneous nature and chemoresistance development. However, the results shown in this manuscript are from computational predictions, and *in vitro* validations, therefore, further preclinical and clinical investigations are warranted to validate SBL-JP-0004, paving the way for potential advancements in the treatment landscape of GC.

## Data Availability

The datasets generated and/or analyzed during the current study are available from the corresponding author upon reasonable request.

## References

[1] Jaroenlapnopparat A, Bhatia K, Coban S. Inflammation and gastric cancer. Diseases. 2022;10(3):35. doi:10.3390/diseases10030035; 35892729 PMC9326573

[2] Sepulveda AR. Helicobacter, inflammation, and gastric cancer. Curr Pathobiol Rep. 2013;1(1):9–18. doi:10.1007/s40139-013-0009-8; 23687623 PMC3655720

[3] Tempera PJ, Michael M, Tageldin O, Hasak S. Gastric cancer due to chronic H. pylori Infection: what we know and where we are going. Diseases. 2022;10(3):57. doi:10.3390/diseases10030057; 36135213 PMC9498082

[4] Ni Y, Low JT, Silke J, O’Reilly LA. Digesting the role of JAK-STAT and cytokine signaling in oral and gastric cancers. Front Immunol. 2022;13:835997. doi:10.3389/fimmu.2022.835997; 35844493 PMC9277720

[5] Khanna P, Chua PJ, Bay BH, Baeg GH. The JAK/STAT signaling cascade in gastric carcinoma (Review). Int J Oncol. 2015;47(5):1617–26. doi:10.3892/ijo.2015.3160; 26398764

[6] Baccili Cury Megid T, Farooq AR, Wang X, Elimova E. Gastric cancer: molecular mechanisms, novel targets, and immunotherapies: from bench to clinical therapeutics. Cancers. 2023;15(20):5075. doi:10.3390/cancers15205075; 37894443 PMC10605200

[7] Lei ZN, Teng QX, Tian Q, Chen W, Xie Y, Wu K, et al. Signaling pathways and therapeutic interventions in gastric cancer. Signal Transduct Target Ther. 2022;7(1):358. doi:10.1038/s41392-022-01190-w; 36209270 PMC9547882

[8] Tran P, Nguyen C, Klempner SJ. Targeting the Phosphatidylinositol-3-kinase pathway in gastric cancer: can omics improve outcomes? Int Neurourol J. 2016;20(2):S131–40. doi:10.5213/inj.1632740.370; 27915478 PMC5169087

[9] Matsuoka T, Yashiro M. The role of PI3K/Akt/mTOR signaling in gastric carcinoma. Cancers. 2014;6(3):1441–63. doi:10.3390/cancers6031441; 25003395 PMC4190549

[10] Johnson Z, Tarantelli C, Civanelli E, Cascione L, Spriano F, Fraser A, et al. IOA-244 is a non-ATP-competitive, highly selective, tolerable PI3K delta inhibitor that targets solid tumors and breaks immune tolerance. Cancer Res Commun. 2023;3(4):576–91. doi:10.1158/2767-9764.CRC-22-0477; 37066023 PMC10103717

[11] Nürnberg B, Beer-Hammer S. Function, regulation and biological roles of PI3Kγ variants. Biomolecules. 2019;9(9):427. doi:10.3390/biom9090427; 31480354 PMC6770443

[12] Rah B, Rather RA, Bhat GR, Baba AB, Mushtaq I, Farooq M, et al. JAK/STAT signaling: molecular targets, therapeutic opportunities, and limitations of targeted inhibitions in solid malignancies. Front Pharmacol. 2022;13:821344. doi:10.3389/fphar.2022.821344; 35401182 PMC8987160

[13] Zhong Z, Wang T, Zang R, Zang Y, Feng Y, Yan S, et al. Dual PI3K/mTOR inhibitor PF-04979064 regulates tumor growth in gastric cancer and enhances drug sensitivity of gastric cancer cells to 5-FU. Biomed Pharmacother. 2024;170:116086; 38159377 10.1016/j.biopha.2023.116086

[14] Guvenir Celik E, Eroglu O. Combined treatment with ruxolitinib and MK-2206 inhibits the JAK2/STAT5 and PI3K/AKT pathways via apoptosis in MDA-MB-231 breast cancer cell line. Mol Biol Rep. 2023;50(3):319–29. doi:10.1016/j.biopha.2023.116086. 36331743

[15] Wu C, Wong AR, Chen Q, Yang S, Chen M, Sun X, et al. Identification of inhibitors from a functional food-based plant Perillae Folium against hyperuricemia via metabolomics profiling, network pharmacology and all-atom molecular dynamics simulations. Front Endocrinol. 2024;15:1320092. doi:10.3389/fendo.2024.1320092; 38435751 PMC10905266

[16] Valdés-Tresanco MS, Valdés-Tresanco ME, Valiente PA, Moreno E. gmx_MMPBSA: a new tool to perform end-state free energy calculations with GROMACS. J Chem Theory Comput. 2021;17(10):6281–91. doi:10.1021/acs.jctc.1c00645; 34586825

[17] Kamli H, Zaman GS, Shaikh A, Mobarki AA, Rajagopalan P. A combined chemical, computational, and *in vitro* approach identifies SBL-105 as novel DHODH inhibitor in acute myeloid leukemia cells. Oncol Res. 2022;28(9):899–911. doi:10.3727/096504021X16281573507558; 34353411 PMC8790134

[18] Gao JP, Xu W, Liu WT, Yan M, Zhu ZG. Tumor heterogeneity of gastric cancer: from the perspective of tumor-initiating cell. World J Gastroenterol. 2018;24(24):2567–81. doi:10.3748/wjg.v24.i24.2567; 29962814 PMC6021770

[19] Al-Rawashde FA, Al-Wajeeh AS, Vishkaei MN, Saad HKM, Johan MF, Taib WRW, et al. Thymoquinone inhibits JAK/STAT and PI3K/Akt/mTOR signaling pathways in MV4-11 and K562 Myeloid leukemia cells. Pharmaceuticals. 2022;15(9):1123. doi:10.3390/ph15091123; 36145344 PMC9504933

[20] Gerds AT, Bartalucci N, Assad A, Yacoub A. Targeting the PI3K pathway in myeloproliferative neoplasms. Expert Rev Anticancer Ther. 2022;22(8):835–43. doi:10.1080/14737140.2022.2093192; 35763287

[21] Stratikopoulos EE, Parsons RE. Molecular pathways: targeting the PI3K pathway in cancer-BET inhibitors to the rescue. Clinic Cancer Res. 2016; 22(11):2605–10. doi:10.1158/1078-0432.CCR-15-2389; 27250929 PMC4896088

[22] Hu X, Li J, Fu M, Zhao X, Wang W. The JAK/STAT signaling pathway: from bench to clinic. Signal Transduct Target Ther. 2021;6(1):402. doi:10.1038/s41392-021-00791-1; 34824210 PMC8617206

[23] Abohassan M, Alshahrani M, Alshahrani MY, Rajagopalan P. Insilco and Invitro approaches identify novel dual PI3K/AKT pathway inhibitors to control acute myeloid leukemia cell proliferations. Med Oncol. 2022;39(12):249. doi:10.1007/s12032-022-01846-1; 36209300

[24] Tobeigei FH, Gahtani RM, Shaikh A, Al Ali A, Kameli N, Kamli H, et al. Computational high-throughput screening and *in vitro* approaches identify CB-006-3; A novel PI3K-BRAF^V600E^ dual targeted inhibitor against melanoma. Oncol Res. 2021;29(5):305–18. doi:10.32604/or.2022.025187; 37305163 PMC10207986

[25] Pandey R, Kapur R. Targeting phosphatidylinositol-3-kinase pathway for the treatment of Philadelphia-negative myeloproliferative neoplasms. Mol Cancer. 2015;14(1):118. doi:10.1186/s12943-015-0388-z; 26062813 PMC4464249

[26] Wolf A, Eulenfeld R, Gäbler K, Rolvering C, Haan S, Behrmann I, et al. JAK2-V617F-induced MAPK activity is regulated by PI3K and acts synergistically with PI3K on the proliferation of JAK2-V617F-positive cells. JAKSTAT. 2013;2(3):e24574. doi:10.4161/jkst.24574; 24069558 PMC3772110

[27] Judd LM, Menheniott TR, Ling H, Jackson CB, Howlett M, Kalantzis A, et al. Inhibition of the JAK2/STAT3 pathway reduces gastric cancer growth *in vitro* and *in vivo*. PLoS One. 2014;9(5):e95993. doi:10.1371/journal.pone.0095993; 24804649 PMC4013079

[28] Nouri Z, Fakhri S, Nouri K, Wallace CE, Farzaei MH, Bishayee A. Targeting multiple signaling pathways in cancer: the rutin therapeutic approach. Cancers. 2020;12(8):2276. doi:10.3390/cancers12082276; 32823876 PMC7463935

[29] Choudhary N, Bawari S, Burcher JT, Sinha D, Tewari D, Bishayee A. Targeting cell signaling pathways in lung cancer by bioactive phytocompounds. Cancers. 2023;15(15):3980. doi:10.3390/cancers15153980; 37568796 PMC10417502

